# Recent developments in waterborne pathogen detection technologies

**DOI:** 10.1007/s10661-025-13644-z

**Published:** 2025-02-04

**Authors:** Usisipho Feleni, Rebotiloe Morare, Ginny S. Masunga, Nontokozo Magwaza, Valentine Saasa, Moshawe J. Madito, Muthumuni Managa

**Affiliations:** 1 Institute for Nanotechnology and Water Sustainability (iNanoWS), College of Science, Engineering and Technology, University of South Africa, Florida Campus, Florida Park 1710, Johannesburg, South Africa; 2 Department of Life and Consumer Sciences, College of Agriculture and Environmental Sciences, University of South Africa (UNISA), Florida Campus, Roodepoort, 1709 South Africa

**Keywords:** Biosensors, Chemiluminescence, Optical biosensors, PCR detection techniques, Water-borne pathogens

## Abstract

Waterborne pathogens find their way into water bodies through contamination of fecal discharge, stormwater run-offs, agriculture and industrial activities, and poor water infrastructure. These organisms are responsible for causing diarrheal, gastroenteritis, cholera, and typhoid diseases which raise an alarming sense on public human health due to the high mortality rate, especially in children. Several studies have indicated that these waterborne diseases can be managed by monitoring pathogens in water using traditional culture-based and molecular techniques. However, these methods have shown several setbacks such as the longer duration for detection and the inability to detect pathogens at low concentrations. Effective management of these diseases requires rapid, sensitive, highly selective, fast, and efficient economic methods to monitor pathogens in water. Since the creation of biosensors, these tools have been applied and shown the ability to detect pathogens at low concentrations. The highlights of biosensor systems are that they are fast, portable, easy to use, highly sensitive, and specific. The capabilities of biosensors have given these tools exposure to be widely applied in detecting pharmaceutical pollutants, pesticides, toxins, residues of detergents, and cosmetics from household activities in soil and water. With such difficulties faced for detecting waterborne pathogens, this review evaluates the effectiveness of technologies for waterborne pathogens detection and their drawbacks. It further highlights biosensors as the current reliable method available for detecting pathogens in water and its future capabilities in sustaining safe potable water.

## Introduction

Water is a necessity for the existence of life. Several human activities from household activities, personal hygiene, recreational purposes, and production of food products to health care activities requires water. So, it is vital that the quality of water is acceptable to meet sustainable healthy living. However, it is not always the case as water has shown to be potential carrier of various contaminants (biological and inorganic water pollutants) which pose a risk to human health. Entry of biological contaminants in water can be through human and animal feces, poor infrastructure of a water system, stormwater floods, and industrial and agricultural waste (Agarwal et al., 2014; Aw & Rose, 2012). Waterborne pathogens can be present in different settings of water bodies and can remain there for longer period if they are not detected and treated. This poses an extreme risk to livelihoods, as humans and animals can simply come in contact with waterborne pathogens. Drinking and physical contact with water contaminated with waterborne pathogens such as bacteria, viruses, protozoa, and helminths result in a high risk to human health (Amini & Kraatz, 2015). In Table 1, variety of diseases affecting human health because of consuming contaminated water. Diseases caused by pathogens in water result in death in severe cases or cause constant health problems. These diseases also create an economic burden due to the expenses spent on treatment. The economy is further burdened by absence of individuals in areas of production due to illnesses. Approximately 1.6 million death cases are reported annually because of waterborne pathogens in the world (Zahedi et al., 2021). Children are the most affected among the annual waterborne mortality cases (Li et al., 2020).

**Table 1:** Human diseases caused by various waterborne pathogens

	Microbial pathogens	Diseases	References
Bacteria	*Vibrio cholerae* *Escherichia coli* *Samonella typhi* *Shigella dysenteriae*	Cholera Diarrhea, gastroenteritis Typhoid Dysentery	Magana-Arachchi and Wanigatunge (2020); Noureen et al. (2022); Shayo et al. (2023)
Viruses	Enteroviruses Hepatitis A and E viruses Adenoviruses	Gastroenteritis Hepatitis Gastroenteritis	McKee and Cruz (2021); Ramírez-Castillo et al. (2015); Weaver et al. (2023)
Protozoa	*Giardia intestinalis* *Entamoeba histolytica* *Cryptosporidium*	Gastroenteritis Amoebiasis Gastroenteritis	Collier et al. (2021); Gupta et al. (2022); Magana-Arachchi and Wanigatunge (2020)

To ease the burden of waterborne diseases, control measures to ensure potable water quality must be created to grant human safety. To minimize and prevent the risk of waterborne diseases, many countries have implemented acceptable limits for waterborne pathogens in drinking water (Kumar et al., 2019a). WHO recommendations for bacterial pollutants in drinking water state that no more than 100 mL of water should contain detectable levels of coliforms and general bacteria. For example, *Escherichia coli* must not be detected in any 100 mL sample of drinking water and can ideally be 126 CFU (colony-forming units)/100 mL for domestic and recreational water (Gunda et al., 2016). Its presence may indicate fecal contamination and potential presence of pathogens. Once pathogen levels are detected to have reached a certain concentration, the water can be confirmed as unsafe for use. The management of diseases caused by waterborne pathogens can be achieved through the identification and quantification of pathogenic microorganisms in water. This measure alerts communities of possible waterborne disease outbreaks. Although these methods detect pathogens that cause diseases in water, the period of detection is a critical factor when preventing or managing disease outbreaks (Wang et al., 2017). Several detection methods have been applied to monitor pathogen levels. Traditionally, microbial detections were done through laboratory-based methods such as phenotype, molecular-based and analytic (Zulkifli et al., 2018).

The culture-based method is described as simple and cost-effective for detecting microbial cells and it is referred to as a golden standard (Li et al., 2020). Culture-based methods commonly use an indicator microorganism that shows pathogen contamination in water. Faecal coliforms are used as indicator microorganisms in water quality analysis, the commonly used indicator is generic *E*. *coli* due to its simplicity to culture and is easily quantified in the laboratory (Bivins et al., 2020; McEgan et al., 2013; Saxena et al., 2015). Other than *E*. *coli*, a diversity of other bacterial genus, viruses and bacteriophages can also be used as indicator organisms in water quality assessment (Gorski et al., 2019). The use of an indicator microorganism is not an attractive method for water quality analysis in risk of waterborne diseases as it takes 18–72 h to detect the presence of coliforms (Hossain & Mansour, 2019). Traditionally, coliform monitoring in the water sample is achieved by the application of culture-based methods, multiple-tube fermentation (MTF), and membrane filtration. These methods are not effective control measures to follow during waterborne disease outbreaks as it is time-consuming, highly laborious, low sensitivity for detection of contaminants present in low concentration and inability to detect non-culturable pathogens (Ahmed et al., 2014; Alhamlan et al., 2015; Li et al., 2020). According to Henry et al. (2015), poor detection of pathogens in low concentration is improved by pre-enrichment of the analyte which draws back to time inefficiency. Despite the disadvantages of this method, this method is still widely applied in microbial monitoring. Culture-based method drawbacks have led to molecular methods as an alternative detection route. Overcoming these challenges, molecular based methods presented a viable strategy in monitoring the waterborne pathogens. The outstanding characteristic of molecular methods over traditional microbiology technique is the ability to detect viable cells that are non-culturable (Deshmukh et al., 2016). Molecular methods are based on the principle of targeting a specific genetic material (DNA, RNA) or protein of the target analyte for the identification and quantification of microbes (Rainbow et al., 2020). This constitute of a diverse group of technologies including polymerase chain reaction (PCR), fluorescent in situ hybridization (FISH), enzyme-linked immunosorbent assay (ELISA), and loop-mediated isothermal amplification method (LAMP) (Agarwal et al., 2014; Alhamlan et al., 2015).

The application of molecular tools in water quality analysis has been widely used with a series of successes in detecting and quantifying waterborne pathogens. These methods are rapid, highly sensitive, and specific; some of these methods can withstand the presence of inhibitors (Wang et al., 2017). However, they also have inheritable disadvantages that hinder the effectiveness of their performance in assessing the microbial quality of water. They require specialized equipment, which can be costly (Li et al., 2020). Additionally, the reproducibility of these methods may be impacted by inhibitors (Farkas et al., 2017). They often require extensive sample pre-treatment and must be operated by highly trained personnel (Saad & Faucher, 2021). Some molecular methods face challenges, such as the similarity of 16S rRNA gene sequences between non-pathogenic and pathogenic strains (Ishii et al., 2013). Additionally, the inability of culture-based and molecular methods to detect pathogens at low concentrations poses a health risk to communities, as waterborne pathogens can remain infectious even at minimal levels (Wu et al., 2020).

The limitations of traditional methods have driven scientists to develop more effective detection techniques that are sensitive, specific, fast, easy to apply, cost-effective, and capable of detecting pathogens at low concentrations for monitoring purposes (Ramírez-Castillo et al., 2015). The emergence of biosensors has addressed hurdles in monitoring pathogens. Biosensors are technological tools that function based on two components, a biological molecule (DNA, antibodies, enzymes, bacteriophage) or artificial biomaterial (aptamers) that recognizes and binds to target analyte and a transducer, i.e., an element that produces a signal once the biomolecule interact with the analyte (Rainbow et al., 2020). In comparison with conventional methods, biosensors detect an analyte of interest without pre-concentration of the sample which takes less time for analysis (Idil & Mattiasson, 2017). They have also shown the character of high specificity and sensitivity, low cost, ease of use and miniaturization, which makes biosensors an attractive detection method of pathogens in water (Jiang et al., 2020). These factors are important in the detection of the pathogens in water because they allow authorities to alert communities about contamination in minimal time and to come up with preventative measures before the contaminated water reaches humans for usage. The technological modelling of biosensors can be optimized to allow improved functionality in different settings of waster analysis (Song et al., 2023). The enhancement of biosensors performance can be achieved through incorporation of nanomaterials.

In many studies, biosensors have been broadly applied in medical diagnostics centres, water, food, and agricultural industries for the detection of pathogens (Bhatt & Bhattacharya, 2019). To strategically manage waterborne diseases outbreaks in communities and to lessen the economic burden for treatment of waterborne diseases, newly developed technologies must be applied to quickly detect the pathogens in water. Culture and molecular-based methods have been attributed to multiple setbacks that affect the effective monitoring of waterborne pathogens. Therefore, the emergence and application of biosensors with the incorporation of nanomaterials have shown great progress in evolving technologies towards efficient pathogen monitoring in water systems. In this review, both traditional and modern methods used in detecting pathogens in water will be evaluated for their efficiency and drawbacks and which technology will be reliable for the detection of waterborne pathogens in cases of urgent waterborne disease outbreaks.

## Culture-based method

The culture-based method is a standard method for analysing microbial presence in water, soil, and food samples. This technique is still in use for the detection of pathogens in water, regardless of the newly developed techniques. Optimization of these methods involves pre-enrichment steps of media and using selective media (Aladhadh, 2023; Foddai & Grant, 2020; Henry et al., 2015). A key feature of culture-based methods is the utilization of selective media. These media are specifically formulated to encourage the growth of target bacteria while simultaneously suppressing the growth of non-target organisms, thereby ensuring more precise detection and isolation of the desired pathogens (Oon et al., 2023). Uzoigwe et al. (2012) conducted a microbiological quality analysis of water samples from dumpsite and non-dumpsite to identify bacteria. In this study different selective media were used for culturing heterotrophic bacteria, fecal coliforms, *Salmonella*/*Shigella*, and *Vibrio* species and the isolates were incubated for 48 h to obtain the results. The results of the study showed that water near the dumpsite is highly contaminated with bacteria as the fecal coliforms quantity ranged from 2.5 × 10^2^ to 4.5 × 10^2^ CFU/mL in water samples near the dump site. The results further showed that *Salmonella*/*Shigella* and *Vibrio* species had high loads in water samples near the dumpsite. It is evident that the method was able to detect pathogen contamination; however, it is time-consuming and laborious as different media had to be prepared.

**Fig. 1:**
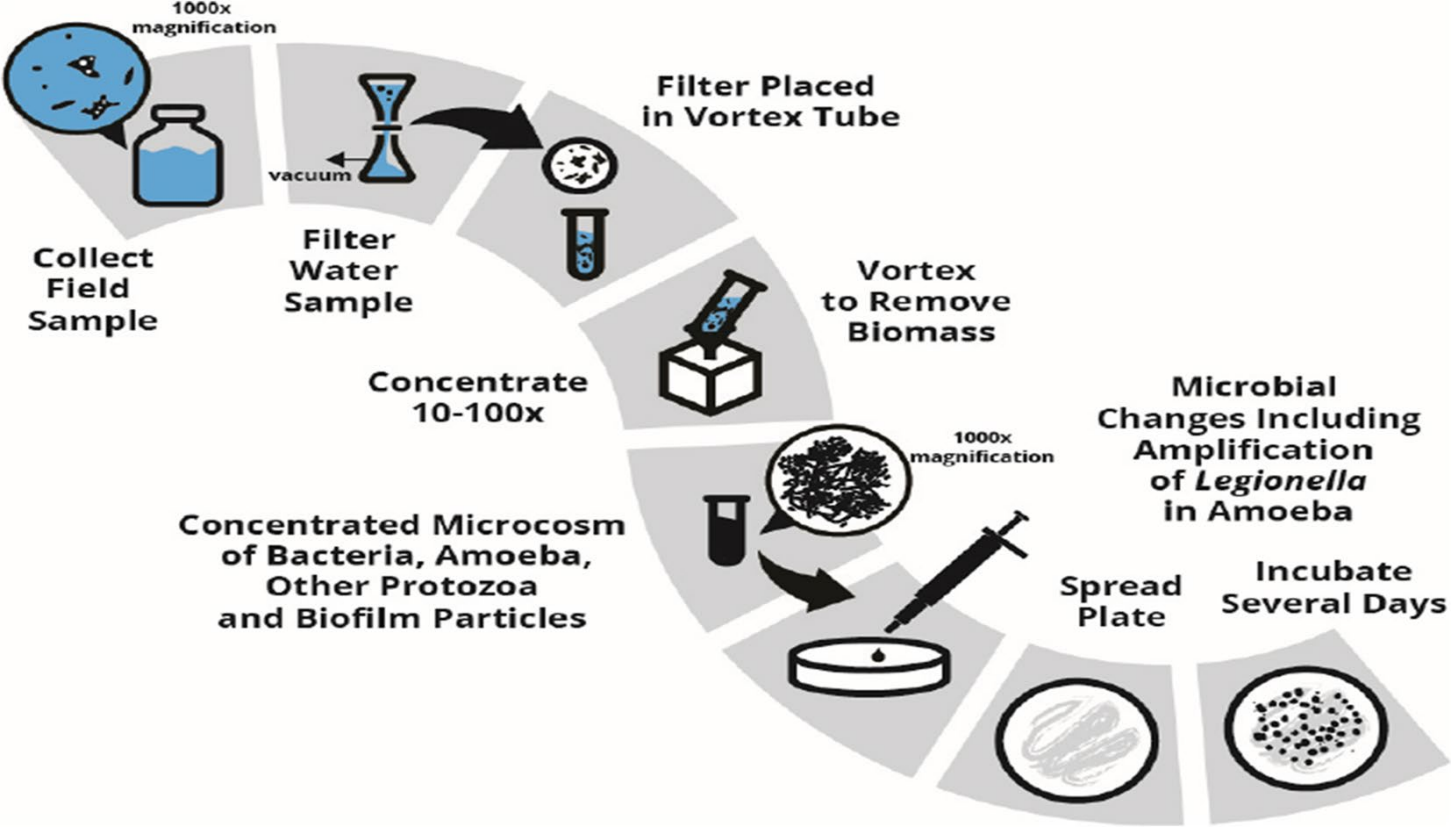
A schematic diagram showing the process of using microbiological methods for monitoring pathogens in water (McCoy & Rosenblatt, 2015)

In Fig. 1, multiple steps of traditional microbiological processes for the detection of pathogens are shown. The methods were used for monitoring *Legionella* which is one of the waterborne pathogens and other microbial pathogens in water. Abdul et al. (2012) have assessed the microbiological quality of drinking water using standard plate count, membrane filtration, thermotolerant coliforms and the most probable number which are culture-based methods. The results of the study have shown that the concentration of total heterotrophic bacteria which was used as an indicator ranged from 1.0 × 10^5^ to 18 × 10^7^ CFU/mL. However, the total number of viable bacteria were too many to count on the plates. This only indicated that the water was contaminated and was unsafe to drink. This shows unreliability of this method, because of limited information of the present microbes including their quantity, strains, and infectivity. Before detecting pathogens, the samples must be filtered, pre-concentrated, and preparation of culture media which can be labour intensive. It is also indicated that the culture-based method takes several days to get results. These limitations render culture-based methods inefficient in determining microbial risk assessment in water. This method may lead to cross-contamination of samples and give false-positive results as it quantitatively reports on only culturable microbes. In another study, Ahmed et al. (2010) have applied the traditional culture-based method to assess the microbiological quality of roof-harvested rainwater by monitoring the concentration of fecal indicator bacteria. The microbes (*E*. *coli*, enterococci, and spore-forming *Clostridium perfringens*) were isolated through membrane filtration technique. The filters were placed on the respective agar medium of each fecal indicator bacteria and incubated for 24 h. The results showed that 83%, 58% and 46% of the 100 tested samples were positive for enterococci, *E*. *coli*, and *C*. *perfringens* respectively. Although the method showed an ability to detect fecal indicator bacteria, it still requires a lot of effort and is time-consuming. Another disadvantage of the application of fecal indicators remains unreliable or brings low confidence in the microbiological quality analysis of water due to no clear relation with pathogens. Lin et al. (2020) conducted a study where traditional culture-based techniques were used to monitor waterborne pathogens in household water purifiers as well as influent and effluent waters. Drinking water was treated through five different purifiers, the membranes of the purifiers were pre-treated, and the supernatant was spread on nutrient agar (NA) and R_2_A agar and incubated to quantify bacteria. The results of the study showed there was no bacterial growth from influent water on nutrient agar plates, but a high concentration was observed on effluent water. This observation can be associated with the microorganisms that were non-culturable on nutrient agar or the growth conditions that were not favourable. Although there was growth in the R_2_A medium, the no-growth on NA makes the traditional culture-based method ineffective in monitoring microbial pathogens in drinking water. It was further observed quantification of microbes from the purifiers ranged from 10^2^–10^6^ CFU/g. The results of the study fulfilled the purpose of the study; however, modern techniques could increase the efficiency of determining microbial concentration on household water purifiers.

### Fecal coliforms as indicators

Fecal coliforms are indicator microorganisms for the presence or absence of pathogens in water which determine the safety of water for drinking and other activities (Maheux et al., 2014). They are used as indicators because they are habitants of the intestinal tract of humans, therefore their presence in water provide indicates that the water is contaminated especially in water distribution systems (Bisimwa et al., 2022; Saxena et al., 2015). They are also used to advance the modelling of infectious disease risks (Sikder et al., 2021). It is recommended that for safe potable water, fecal coliforms should be at zero concentration per 100 mL of water (Agarwal et al., 2014; Zulkifli et al., 2018). The reliability of using coliforms for determining the number of pathogens is not of high confidence value as there is no direct link between the number of coliforms and pathogens (Odonkor et al., 2013). Saingam et al. (2020) conducted a study to assess the microbiological quality of marine water by monitoring fecal indicator bacteria (FIB), pathogens, and the overall microbial community. FIB was identified using membrane filtration techniques. This method was also applied to detect pathogens, which were subsequently cultured on their specific enriched growth media. The study found that the concentration of *E*. *coli* was significantly higher (132 CFU/mL) compared to enterococci and *Clostridium perfringens* (18 CFU/mL and 21 CFU/mL, respectively). Additionally, the analyzed pathogens (*Salmonella*, *Listeria monocytogenes*, *Campylobacter*) were not detected in any of the water samples, except for *Vibrio parahaemolyticus*, which was found in high concentrations. This pathogen was also detected at high levels using molecular methods. When comparing the detection of pathogens and FIB, the study revealed no correlation between the two, indicating that the presence of coliforms in water does not accurately reflect the concentration or identification of pathogens. Thus, this method does not address the risks posed by waterborne pathogens, which can lead to serious health issues. Coliforms are typically detected through cultivation on differential media under controlled conditions, followed by identification and quantification (Molina et al., 2015). Standard microbiological methods for coliform monitoring can be time-consuming, often requiring more than 24 h for accurate identification (Offenbaume et al., 2020), which limits their effectiveness as an early warning system for pathogen contamination. Common techniques for cultivation and quantification include multiple fermentation, membrane filtration, and the most probable number method. Jacinta et al. (2015) used multiple tube fermentation and the most probable number methods to determine fecal coliforms from various drinking water sources. In their study, some samples that showed no visible gas production in the presumptive method were re-incubated for an additional 24 h. Afterward, two more tests were performed using specific growth media, and biochemical analysis was conducted for further confirmation. The results showed a high prevalence of *E*. *coli* (71.4%), followed by *Klebsiella pneumoniae* (14.3%) and *Enterobacter aerogenes* (14.3%). These findings indicated that the water was unsafe for domestic use. Despite the detection of fecal coliforms, this method is not reliable for determining pathogen concentrations, as no direct relationship between the two was found. The multiple fermentation step used in this study was labour-intensive and time-consuming, especially in the presumptive stage, where tubes had to be re-incubated. To improve the efficiency of traditional methods, molecular techniques can be integrated for more accurate quantification of coliforms and pathogens.

#### Multiple fermentation tube

Multiple fermentation tube (MTF) is a microbiological procedure which is performed in three steps a presumptive, confirmation, and completion steps for monitoring microbial presence in water samples (Zulkifli et al., 2018). Post presumptive results are indicated by gas production and acid formation (Deshmukh et al., 2016). The three MTF steps were followed in a study done by Jacinta et al. (2015) for detection of coliforms in different drinking sources, in all the steps gas production was observed indicating the presence of microorganisms. Rahman et al. (2013) used this three steps method to identify and quantify *E*. *coli* in drinking water, where formation of gas indicated bacterial growth and further used another microbiological method to confirm the presence of *E*. *coli*. Formation of gas which is observed as bubbles in liquid medium and acid as well as colour change of lactose peptone culture medium to yellowware a positive indication of bacterial presence (Bhattacharyya, 2018; Kuo et al., 2020). In a study by Shen et al. (2019), MTF was used determine the presence of coliforms from 120 water samples, from the overall samples only 23 coliforms were detected between 2 and 3 days.

Bhattacharyya (2018) has indicated in the study that MTF can give inaccurate identification of bacteria; therefore, it was necessary for this method to be coupled with other techniques. The efficiency of this method was evaluated against molecular method polymerase chain reaction (PCR) in a study conducted by Fard et al. (2019). The results of the study showed that MTF detected only 53.3% in eight samples and 40% in six samples of coliforms and *E*. *coli* respectively over 3–4 days, while PCR showed higher detection efficiency in a short period of time. Although the method is easy to follow practically and inexpensive due to that it does not involve specialized equipment, MTF is labor intensive due to the series of dilutions in the process (Rompre et al., 2002). The series of dilution and labour intensity leads to long response time which hinders the strategic goal of microbial risk alertness in period of water disease outbreaks. Another setback of this method is a lack of precise quantification for the microbes in water sample which makes it less desirable for controlling water risks.

#### Membrane filtration

Microbiological laboratories employ membrane as an alternative microbial detection method to multiple fermentation tube. Membrane filtrations have been used diversely for enumerating and isolating pathogens in medical applications, water and food analysis, gas purification, and sterilization (Lee et al., 2007). Membrane filtration is used in many countries as a standard method for assessing microbial population in drinking water (Rompre et al., 2002). In the process of membrane filtration, pathogens in water samples are detected and quantified by employing sterile micro-filter of 0.45 µM to trap microbes (Shen et al., 2019). Once the microbes are filtered, the filter is placed on selective media for microbial cultivation (Alhamlan et al., 2015). Usually, quantification of microbes by this method takes 24–48 h, after visible yellow colonies appearance on the medium (Shen et al., 2019). Dufour et al. (1981) enumerated *E. coli* from freshwater by passing water samples through 0.45 µM and the filters were transferred on pads saturated with urea substrate for urease test. The yellow colonies on the pads were verified as *E*. *coli* by further culturing on nutrient agar and Simmons citrates agar followed by incubation for 24 h. The method further required biochemical test to confirm the presence of *E*. *coli*. This shows that membrane filtration is laborious and takes time to quantify microbes. The efficiency of this method can be affected by cross-contamination during filtration process, inhibitory substances, and turbidity of water sample which do not allow effective filtration (Kuo et al., 2010). Membrane filtration was used in a study by Liu et al. (2013), to detect *Cronobacter* in drinking water. The results of the study indicated that the method was specific with all *Cronobacter* strains having blue-green colonies on growth medium. Microfiltration and ultrafiltration processes can assist with removing microbes from water samples due to their small pore size, therefore making the water to be potable (Zulkifli et al., 2018). In a study by Sinclair et al. (2018), commercial cationic polyether sulfone (PES) microfiltration (MF) membranes were developed with the aim of reducing waterborne viruses from drinking water. The results of that study showed the developed polymer microfiltration membranes reduced 99.9% of MS2 bacteriophage viral load.

Kuo et al. (2010) have stated that the pathogen detection efficiency of this method can be affected by the turbidity of water, presence of toxicants, and cross contamination while filtering can occur. Like, the multiple fermentation tube the membrane filtration is also time consuming, therefore it is unreliable to use this method for pathogen quantification for urgent water analysis. Despite the weaknesses of microbiological methods, these are still commonly used in laboratory to identify and quantify pathogens and are often complemented with molecular techniques for more accurate results.

## Molecular methods

The limitations of culture methods have led to alternatively using molecular technologies which have evidently shown rapid, highly sensitive, and specific determination of pathogens (Alhamlan et al., 2015). This method is also outstanding on the ability to culture viable but non-culturable microbes which their presence and amount in water can be easily passed by microbiological methods (Dungan et al., 2012). Molecular method consists of a group of techniques which use nucleic acid either deoxyribonucleic acid (DNA) or ribonucleic acid (RNA) or protein to detect the target analyte. These methods are classified based on their distinctive procedures and materials used. Enzyme-linked immune sorbent assay (ELISA), Western blotting (WB), immunofluorescence assay (IFA), hemagglutination inhibition assay (HAI), polymerase chain reaction (PCR), nucleic acid amplification tests (NAATs), fluorescent in situ hybridization (FISH), and loop-mediated isothermal amplification (LAMP) are molecular methods which some of them are described in this section along with their capabilities and weaknesses in the output of detecting pathogens.

### Enzyme-linked immune sorbent assay

Enzyme-linked immune sorbent assay is an innovative immunological technique which permits identification of pathogens and non-biological substance by using antibody and antigen complexes (Ramírez-Castillo et al., 2015). This method relies on specificity of antibody to antigen to give quantitative measure of the targeted analyte. Xi et al. (2007) have conducted a study where *E*. *coli* in drainage of wastewater was detected by ELISA with a detection limit ranging from 10–6 × 10^4^ CFU/mL. The antigen–antibody reaction of ELISA for rapid and sensitive detection of *E*. *coli* was achieved by pre-treatment of *E*. *coli* cells by ultrasonic instrument and optimisation of ELISA experimental conditions.

Microbial analysis in water require process which give results in short response time, due to that many water sources are used for household supply on daily basis. Lengthy process can have effect on identification and quantification of microbial population, therefore placing human’s lives at a potential risk of diseases. ELISA technique which is laborious due to several steps such as blocking, washing, incubation of antibodies, and substrate can be ineffective for urgent microbial quantification, due to the time spent on performing the steps (Byrne et al., 2009). To enhance the efficiency of molecular detection, ELISA can be coupled with PCR which increases detection time and respond in short period. Even though the combination works efficiently, these methods are still greatly affected by inhibitors in complex matrices of water samples (Bhattacharyya, 2018).

### Fluorescent in situhybridization

The use of molecular methods for pathogen detection remains a cornerstone in water analysis. Ongoing research and the development of innovative molecular technologies are essential to achieving higher standards of detection. These advancements aim to reliably quantify microbial populations, a critical component for assessing water quality. Reliable water quality assessments ensure the safety of drinking water and enable authorities to implement necessary remedial actions when risks are identified. One such method, fluorescent in situ hybridization (FISH), facilitates both qualitative and quantitative detection of microbes in water samples. FISH works by hybridizing fixated microbial cells or rRNA oligonucleotides onto specific probes on a glass slide, followed by visualization using epifluorescence or confocal laser microscopy (Alhamlan et al., 2015; Ramírez-Castillo et al., 2015). This visualization provides quantitative information about the detected pathogens. Kuo et al. (2020) demonstrated the efficacy of FISH for detecting four coliform bacteria—*E*. *coli*, *Klebsiella pneumoniae*, *Enterobacter aerogenes*, and *Citrobacter freundii*—from simulated and domestic water samples. The study optimized FISH conditions, revealing that 3% paraformaldehyde was the ideal fixation compound for all coliforms, as evidenced by the fluorescence intensity and sensitivity. The optimized FISH method enabled the detection of coliforms within 4 h, a significant improvement over traditional microbiological methods, which required 1 to 2 days for detection. Despite its advantages, FISH has inherent limitations. These include the need for pre-treatment steps and its relatively low sensitivity (Deshmukh et al., 2016). Pre-treatment steps can delay pathogen detection, which is critical during urgent waterborne disease outbreaks. Additionally, pre-treatment may increase the concentration of inhibitors, adversely affecting pathogen detection outcomes. Low sensitivity poses another significant challenge, as some pathogens are present in water at very low concentrations. This limitation can undermine the objective of achieving rapid and accurate pathogen detection in water analysis. While molecular technologies like FISH have proven useful for monitoring waterborne pathogens, they are not without drawbacks. To address these limitations, innovative analytical methods such as biosensors have emerged. These technologies demonstrate improved performance in detecting waterborne pathogens and hold promise for overcoming the challenges associated with traditional molecular methods.

### Loop-mediated isothermal amplification

Molecular detection methods have been expanding with the goal to achieve rapid detection in a short space of time, simplicity, and low expenses for operations. In the year 2000, LAMP was invented as an improved molecular method for the detection of pathogens (Yang et al., 2018). The LAMP method allows for the amplification of genes in a simpler manner compared to PCR-based methods. Unlike PCR, the sensitivity of LAMP to the target DNA sequence is unaffected by the presence of non-target DNA and inhibitors (Inácio et al., 2008). This innovative molecular tool amplifies genes through auto-cycling activity using strand-displacing DNA polymerase and six specifically designed primers (Ghosh et al., 2015). The six set of primers consist of two forward inner primers, two backward inner primers, and two outer primers which each recognizing its distinct sequences on the target DNA ensuring high specific detection (Vielba-Fernández et al., 2019). Bosward et al. (2016) employed LAMP for the detection of *Streptococcus agalactiae*, and the results demonstrated that this method was highly specific to the targeted *S*. *agalactiae* strain, with non-target strains remaining undetected. Additionally, the method showed sensitivity by detecting the targeted DNA at a low concentration of 20 pg per reaction. In another study, Tongphrom et al. (2018) utilized LAMP in combination with the most probable number (MPN) method to detect and quantify total coliforms and *E*. *coli* in water and vegetables. The lacZ-LAMP and uidA-LAMP primers developed for this study were specific to all tested coliforms, with the latter also detecting 30 strains of *E*. *coli* and *Shigella* spp. with positive results. The LAMP method proved to be more sensitive than traditional PCR, with a detection limit of just 1 colony-forming unit (CFU) per reaction for *E*. *coli*. The MPN-LAMP method successfully detected *E*. *coli* at 1 CFU/100 mL in water and 5 CFU/g in vegetables.

Martzy et al. (2017) compared the sensitivity and specificity of LAMP and quantitative PCR (qPCR) for detecting *Enterococcus* spp. at three different DNA concentrations (1 ng, 10 pg, and 0.1 pg per reaction). The results revealed that LAMP could detect the targeted *Enterococcus* spp. at a very low concentration of 0.1 pg DNA per reaction. However, LAMP also detected non-targeted pathogens, which was not observed with qPCR. This discrepancy may be due to high sequence similarity between species in the amplified gene fragment. One of the key operational advantages of LAMP is that it does not require a thermal cycler for gene amplification, allowing for a faster process and a constant temperature throughout amplification (Li et al., 2020). The amplification can be performed with a simple setup, such as a water bath or heat block, at a constant temperature typically between 60 and 65 °C (Zanoli & Spoto, 2012). Additionally, the amplification products can be visualized by incorporating a dye, eliminating the need for electrophoresis. Njiru et al. (2008) demonstrated that including loop primers in the LAMP assay at 62 °C reduced the reaction time from 50 to 25 min, enhancing sensitivity for detecting *Trypanosoma brucei rhodesiense*. Zhang et al. (2013) used several detection methods to analyze LAMP-amplified products of *Streptococcus* *suis* serotype 2, including gel electrophoresis, SYBR green, turbidity, calcein, and hydroxynaphthol blue detection. SYBR green provided the highest sensitivity, with a detection limit of 7.16 copies per reaction. These detection methods proved to be more reliable than electrophoresis. Furthermore, the primer sets used in LAMP were specific to *S*. *suis* serotype 2, yielding positive results. The simplicity of the LAMP method, without the need for complex equipment like thermal cyclers, makes it a viable option for on-site testing, with portable detection materials (Martzy et al., 2019). This method present advantages which can be effective in managing risk of water pathogen contamination to prevent waterborne outbreaks in communities especially in a resource limited area. It has also been applied as a strategy for determining and managing biological risks in food quality analysis.

### Polymerase chain reaction

PCR is widely recognized as an alternative to culture-based methods, offering rapid, sensitive, and highly specific microbial detection. This three-step process—denaturation, annealing, and extension—is performed at specific temperatures using a thermocycler (Ramírez-Castillo et al., 2015). The technique amplifies target gene(s) of interest, but its reliance on specialized, costly laboratory equipment and the need for skilled operators can make it impractical for resource-limited communities. Primers used in PCR can be tailored to ensure precise detection of target genes (Haramoto et al., 2018). Additionally, optimal reaction mixtures play a crucial role in achieving accurate results (Ramírez-Castillo et al., 2015). Despite its utility, conventional PCR has notable limitations, such as lengthy response times, the necessity for post-PCR analysis, and a higher risk of contamination. Furthermore, its dependence on complex, laboratory-based equipment restricts its applicability for on-site analysis. To address these challenges, various advanced PCR technologies have been developed, facilitating efficient, rapid detection of pathogens across environmental, medical, and food analysis domains.

#### Advances in PCR: multiplex PCR

The response time of traditional PCR has been significantly improved with the advent of multiplex PCR (mPCR). This method enables simultaneous amplification of multiple target genes within a single reaction tube by using specific primers designed for various pathogens (Alhamlan et al., 2015). However, the design of these primers can introduce delays in the process (Velusamy et al., 2010). Park et al. (2011) evaluated the sensitivity and specificity of primers in mPCR using microbial strains and three primer sets targeting *Campylobacter* ssp., *Salmonella* ssp., and *E*. *coli*. While primers for *Campylobacter* and *E*. *coli* showed high specificity, those for *Salmonella* formed primer dimers. Despite this, mPCR reduced detection time to 4 h, compared to the 48 h required by culture-based methods.

Similarly, Fard et al. (2019) demonstrated the time-saving benefits of mPCR. Detection of coliforms and *E*. *coli* took approximately 10 h, significantly faster than the traditional Most Probable Number (MPN) method, which required 72 h. High sensitivity was noted, with nearly all 15 water samples testing positive for the target bacteria. Beyond bacterial detection, mPCR has proven effective in identifying other microbial pathogens. Marangi et al. (2015) applied mPCR to detect zoonotic protozoan genes of *Giardia duodenalis* and *Cryptosporidium parvum*. Using DNA from water and mussel samples, mPCR successfully detected oocyst numbers ranging from 10 to 64 per 5 µL. Among 119 water samples, *G*. *duodenalis* was detected in 10, *C*. *parvum* in 18, and both pathogens in 6 samples. These studies highlight mPCR’s ability to reduce detection times, enhance sensitivity, and extend its application to diverse matrices, making it a valuable tool in pathogen detection.

#### Quantitative PCR

Quantitative PCR (qPCR) is widely utilized to quantify pathogen concentrations across various water systems by leveraging a standard curve with known gene copy numbers to estimate the initial concentration of target genes (Crane et al., 2018). For instance, Fumian et al. (2013) demonstrated the application of qPCR in wastewater treatment plants by quantifying viral concentrations ranging from 2.9 × 10⁶ gcL⁻^1^ to 1.81 × 10^3^ gcL⁻^1^ for Human adenovirus, polyomavirus, Norovirus genogroup II, and Human astrovirus. Similarly, Lin and Singh (2015) assessed viral hazards in the Umgeni River, a critical source for domestic and recreational water use, by quantifying adenovirus, rotavirus (RV), and enterovirus (EV) using qPCR. qPCR has also proven superior to culture-based methods for detecting pathogens such as *Legionella* species. Ditommaso et al. (2016) compared the two approaches for analyzing 86 samples from tap water and dental unit waterlines. While *Legionella* was detected in only six samples using culture methods, qPCR identified the pathogen in all the samples tested, underscoring its sensitivity and reliability.

##### Challenges and limitations of qPCR

Despite its advantages, qPCR faces certain limitations, particularly the presence of inhibitors in water samples that can hinder effective results (Mull et al., 2013). Wolf-Baca and Siedlecka (2019) demonstrated accurate qPCR detection of *Legionella* ssp., *Legionella pneumophila*, and *E*. *coli* from hot tap water. However, one sample showed high contamination levels, with color substances (possibly iron and manganese) acting as inhibitors and preventing successful DNA detection. To address the challenges associated with qPCR, further advancements are needed to enhance its ability to detect and quantify pathogens accurately. Improving sample preparation methods, developing more robust protocols to mitigate the effects of inhibitors, and optimizing reaction conditions can make qPCR even more reliable for diverse applications.

##### Optimization of qPCR: microfluidic quantitative PCR

**Fig. 2:**
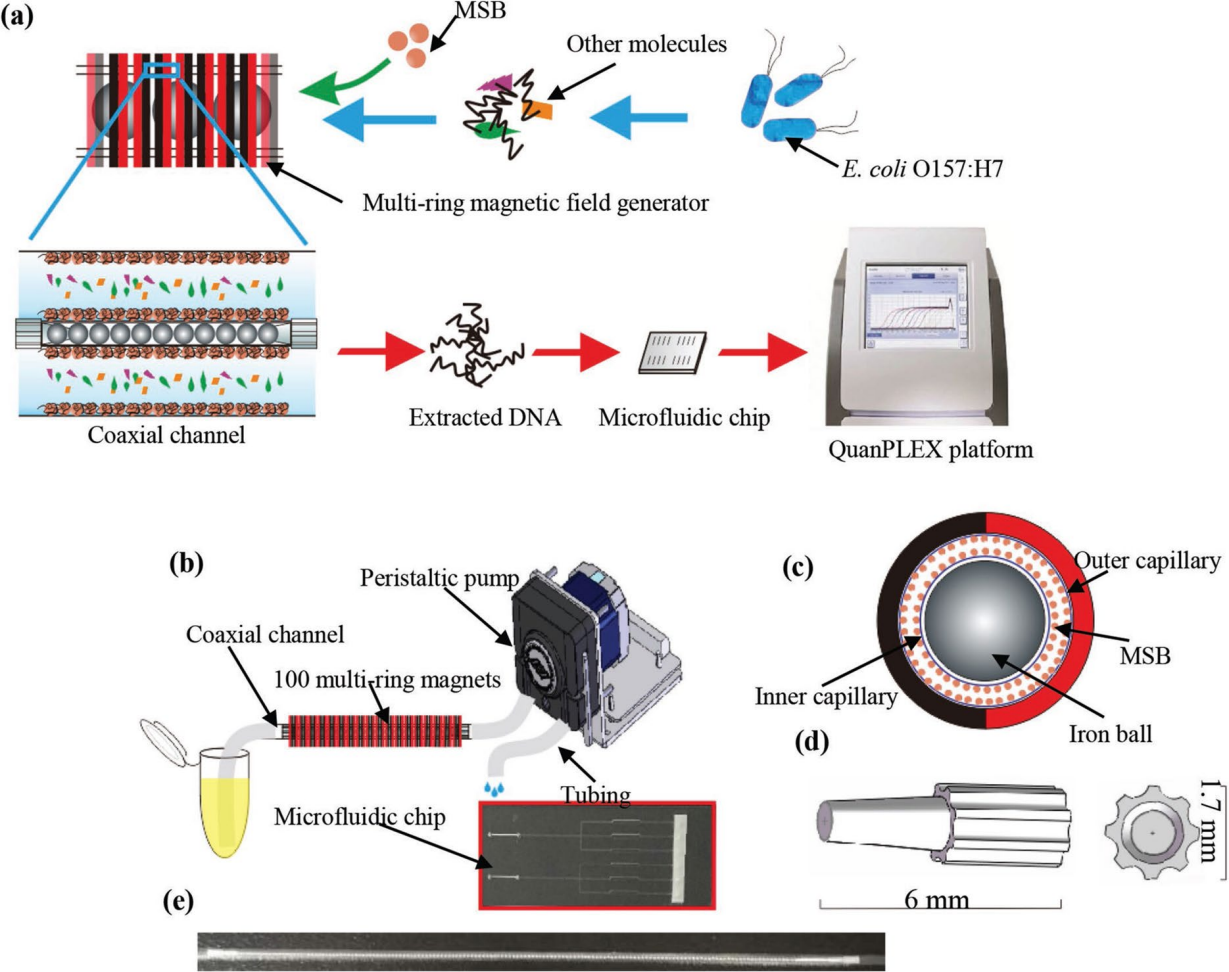
**a** Schematic of the effective and sensitive method of nucleic acid extraction using coaxial channel with magnetic silica beads; **b** schematic of the DNA extraction device; **c** schematic of the coaxial channel with the magnetic silica beads; **d** schematic of the aligner fabricated by the 3D printer; and **e** the coaxial channel. QuanPLEX, Each-Reach, Qingdao, Shandong, China. *MSB* magnetic silica beads (Zhang et al., 2018)

Research has focused on optimizing qPCR through the development of microfluidic quantitative PCR, a method that has demonstrated rapid quantification in various studies. This advanced technique enables the simultaneous detection of multiple genes from target pathogens in complex environmental samples. It provides a quantitative overview of multiple pathogens in a single reaction, making it a convenient tool for assessing the severity of pathogen risks in water quality. Ishii et al. (2013) utilized microfluidic qPCR to detect and quantify multiple viral and bacterial genes in effluent sewage water and human fecal samples within a single assay. Remarkably, this method successfully detected and quantified gene molecules at concentrations as low as 2 copies µL^−1^ in environmental samples. Similar findings were reported by Byppanahalli et al. (2015), who applied microfluidic qPCR to detect virulence factor genes of generic *E*. *coli*, enteropathogenic *E*. *coli* (EPEC), Shiga toxin-producing *E*. *coli*, and *Shigella* from beach water. The study demonstrated that the quantities of gene molecules detected by microfluidic qPCR were consistent with those obtained through traditional qPCR, as illustrated in Fig. 2. These results highlight that microfluidic qPCR is less time-consuming compared to traditional qPCR, which is limited to detecting single or a few pathogens per reaction. This efficiency, combined with its ability to process complex samples, makes microfluidic qPCR a promising advancement for pathogen detection in environmental monitoring.

#### Digital droplet PCR

The challenge of inhibitors effect on qPCR efficiency has led to modification of PCR to achieve rapid detection. Digital droplet PCR (ddPCR) is the advanced technological form of PCR which quantify genes without interference by inhibitory substances in the reaction mixture Singh et al. (2020). This technique allows absolute quantification of DNA template by separating reaction mixture into multiple thousands water-in-oil droplets prior thermal cycling (Cremonesi et al., 2016). After amplification, detection of target gene is determined by fluorescent of each droplet which indicates either positive or negative reaction (Ramírez et al., 2018). The simplicity of this method over qPCR is that it does not require calibration curves for quantification of number gene copies (Xu et al., 2020). This method has been successfully used to detect bacteria and viruses in many studies. Singh et al. (2017) have shown that ddPCR was able to detect *Salmonella* tar gene with the lowest concentration of 2 gene copies per single PCR in water, therefore showing sensitivity as compared to qPCR which detected 20 CFU per PCR. Digital droplet PCR have been applied in many fields of studies, but it is rarely applied for environmental analysis (Ma et al., 2020). As such, there is limited information of efficiency and setbacks of this method in application to detect waterborne pathogens. Developments of molecular studies leads to methods which simply overcome the challenges experienced with PCR for detection of pathogens in water. Several PCR techniques, although they are enhanced regularly, they still present limitation such as inability to differentiate viable from non-viable cells and detection of pathogens present in water at low concentration.

## Biosensors

Research across scientific fields has increasingly focused on developing detection methods that efficiently identify and quantify microbial pathogens, toxins, and metals in water. The advent of biosensors has led to numerous successes in their application for environmental analysis, medical diagnostics, food safety assessments, and security purposes (Srinivasan, B. & Tung, 2015; Chadha et al., 2022; Bhatia et al., 2024). The concept of biosensors originated in 1962 when Leland Clark used them to measure glucose in biological matrices (Vigneshvar et al., 2016). Biosensors are often described as innovative analytical tools that can effectively detect specific biological substances in a medium and generate an indicative signal (Ali et al., 2020). The specific recognition model of biosensors resembles the lock-and-key principle of enzyme–substrate complex, which renders them to be highly specific to the target analyte. In addition to the high specificity, they offer wide range of important characteristics that makes them a reliable tool for detection of waterborne pathogens. They are highly sensitive, easy to use, relatively cheap and the response time is short (Lukyanenko et al., 2019). Beyond the efficient performance, some biosensors are designed as a small portable tool, which offers the advantage of an on-site detection (Petronella et al., 2022). It is observed that even if some biosensors are built for on-site pathogen monitoring, they inherit a challenge of poor specificity and selectivity upon conducting on-site assessment (Hui et al., 2022). In a study by Wu et al. (2021), an investigation was done to test the efficiency of a paper-based assay by immobilizing lycopene and whole cell biosensor on paper with the aim of developing portable detection devices. So far, the biosensor research area is accomplishing simplicity in detection of pathogens with high accuracy, which put ease on strategies of managing disease outbreaks.

Biosensors have found extensive use in water quality analysis, particularly in wastewater management, recreational, household, and industrial water systems. Their role in water quality assessment is to provide valuable information about the presence and concentration of pathogens and non-biological contaminants (e.g., metals, pesticides) that pose water safety risks. In this section of the review, we will discuss biosensors, including their structural design, the different classes of biosensors, their applications in various fields, and their use in the water sector and other scientific disciplines.

## Biosensor design

**Fig. 3:**
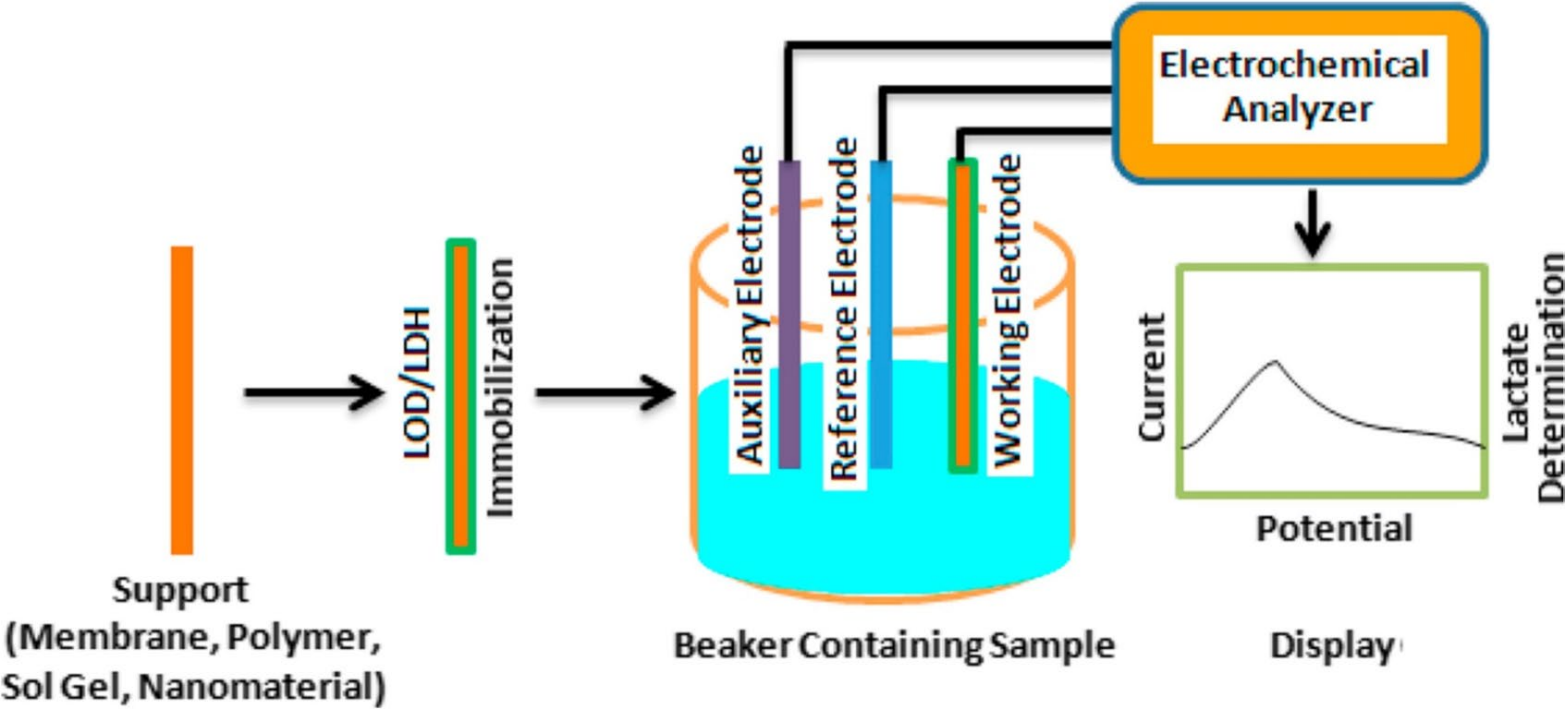
Fundamental structure of biosensor, showing the basic components of biosensor (Rathee et al., 2016; Huang et al., 2017)

The fabrication of biosensors is a crucial aspect that determines the effectiveness of a functioning biosensing system. The core structure of a biosensor consists of a biological recognition component and a transducer element. A basic biosensor model, including these components and the mechanisms involved in analyte detection, is depicted in Fig. 3. The figure also shows a signal processor, which displays the output signal from the transducer after the biorecognition reaction occurs. Both the recognition element and the transducer are essential for the biosensor’s functionality. When the biorecognition component identifies a specific target analyte, the transducer generates an electrical signal that measures the analyte’s concentration. The signal reflects either an increase or decrease in the analyte concentration, thus providing quantitative data. A key factor in designing an efficient biosensor is the proper immobilization of the bioreceptor on the surface of the transducer.

It is crucial to take in consideration the key characteristics when designing a well-functioning biosensors. Adding to high specificity, selectivity, and reproducibility, a well-built biosensor must be able to withstand environmental interferences during the process of detection (Naresh & Lee, 2021). Stability of a biosensor gives an opportunity of to be re-used and ensures accurate results, which validates this technique as cost-effective. This is important when monitoring waterborne pathogens in low income areas due low expenditure while achieving the strategy of managing waterborne diseases. Therefore, designing of biosensors should involve an on-going research which aims at achieving optimum monitoring of target analyte, in this case waterborne pathogens.

Over the years, the modification of biosensors has advanced significantly, driven by ongoing scientific research, leading to faster detection of both biological and non-biological materials. These devices now incorporate cutting-edge technologies, such as nanomaterials and conducting polymers (Kumar et al., 2019b). Conducting polymers, such as poly(3,4-ethylenedioxythiophene) polystyrenesulfonate (PEDOT: PSS), polypyrrole (PPy), and polyaniline (PANI), are integrated during the fabrication process to facilitate the easy immobilization of the biorecognition element (Spychalska et al., 2020). The inclusion of conducting polymers enhances the sensitivity of biosensing systems. These materials are particularly attractive due to their excellent electrical conductivity, a key factor in the effective performance of biosensors (Ramanavicius & Ramanavicius, 2020). Nanomaterials play a crucial role in biosensing systems by improving the immobilization of bioreceptors on the transducer’s surface, thereby enhancing the transducer’s response (Zeng et al., 2016). This is primarily due to the large surface area of nanomaterials and their ability to boost the electron transfer rate on the transducer surface (Batool et al., 2019). As a result, the performance of sensors is significantly improved. Nanomaterials also contribute to enhancing biosensor features such as miniaturization, portability, and rapid signal response (Batool et al., 2019). These innovative, nano-sized particles have become integral to biosensor design, enabling faster detection and contributing to the miniaturized and portable nature of modern biosensors (Willner & Vikesland, 2018). Nanomaterials have been widely used in various biosensor applications, including food safety, environmental analysis, and therapeutic drug delivery (Mansuriya & Altintas, 2020). Common nanoparticles used in biosensor design include gold nanoparticles (AuNPs), gold nanorods (AuNRs), graphene oxide (GO), quantum dots (QDs), and carbon quantum dots (Sanati et al., 2019; Wang et al., 2020). Biosensors can be classified based on their components and functionality. The transducer elements of biosensors can be categorized into electrochemical, optical, and mass-based classes, depending on the type of transducer used. Additionally, biosensors can be classified according to the type of biorecognition molecule they use, such as enzyme-based, immuno-based, cell-based, tissue-based, and nucleic acid-based biosensors (Nigam & Shukla, 2015).

### Electrochemical sensors

The electrochemical biosensor was the first type of biosensor to be developed, initially created for glucose measurement (Vigneshvar et al., 2016). These biosensors operate by detecting electrochemical changes that occur during a biorecognition event, sending signals to the detecting electrode (Idil & Mattiasson, 2017). Since their inception, electrochemical biosensors have expanded rapidly, gaining significant application in detecting both biological and non-biological substances. They are widely used due to their ease of use, low cost, rapid detection time, potential for miniaturization (Wu et al., 2020), low energy consumption (Pires et al., 2014), and their high accuracy and sensitivity (Kaya et al., 2021). In pathogen detection, the target analyte binds to the biorecognition element on the modified electrode, while other analytes remain undetected. The effectiveness of a biosensor largely depends on the choice of the biorecognition element and its method of immobilization onto the transducer (Wu et al., 2018). The functionality of electrochemical biosensors is determined by changes in the signal at the electrode–electrolyte interface. These signal changes can manifest as changes in current, voltage, or resistance, providing both quantitative and qualitative data on the target analyte (Zhang et al., 2019). As shown in Fig. 3, when bacterial pathogens are attached to a magnetic screen-printed carbon electrode, a current change occurs, while the potential remains constant. A properly functioning electrochemical biosensor consists of a three-electrode system: the working, counter, and reference electrodes (Sanati et al., 2019). The working electrode is activated when the target analyte is detected, the reference electrode facilitates oxidation/reduction reactions, and the counter electrode helps to manage the flow of electrical current through the reference electrode (Pires et al., 2014). Various types of electrodes are commonly used to modify electrochemical biosensors, including carbon paste electrodes (CPE), glassy carbon electrodes (GCE), platinum electrodes, gold electrodes, and screen-printed electrodes (SPE) (Rao et al., 2006). The development of electrochemical biosensor detection methods has grown significantly, with the goal of using these tools for the rapid detection of target analytes in the environment. The design of these biosensors is crucial as it directly affects their ability to detect analytes. The fabrication of electrochemical biosensors involves the integration of different basic transducer types, such as amperometry, conductometric, impedimetric, potentiometric (Deshmukh et al., 2016), and voltametric, which together form the different classes of electrochemical biosensors (Li et al., 2020).

### Amperometric immunosensors

Amperometric-based sensors generate a signal when oxidation and reduction occur at the electrode interface in the presence of electrochemically active analytes (Lui et al., 2009). In a study by Rao et al. (2006), a disposable amperometric immunosensor using screen-printed electrodes was developed to detect *Vibrio cholerae* through an indirect sandwich ELISA method. The immunosensor consisted of a working electrode, an Ag/AgCl reference electrode, and a platinum rod as a counter electrode. In the study, rabbit anti-Vibrio cholerae IgG was employed as the capturing antibody for the detection of *V*. *cholerae* antigens using an indirect sandwich ELISA system. The capturing antibodies were immobilized on a solid-phase electrode (SPE) through physical adsorption. The system was then sequentially incubated with *V*. *cholerae* antigens, followed by mouse serum as the revealing antibody, and a rabbit anti-mouse alkaline phosphatase (ALP) conjugate to complete the detection process *V*. *cholerae* was detected at 10^5^ cells/mL within 55 min from a 5 µL sample volume. A similar study by Sharma et al. (2006) developed an amperometric immunosensor for detecting *V*. *cholerae* in various water sources. The antibodies used in this study were highly specific to *V*. *cholerae*, showing no reactivity with other bacterial strains. The immunosensor demonstrated high sensitivity, with detection limits of 80 CFU/mL in sewer and tap water, and 8 CFU/mL in groundwater and seawater. The detection time in this study was comparable to the one by Rao et al. and both studies showed that the amperometric immunosensors could detect *V*. *cholerae* much faster than traditional methods, which typically require longer detection periods. In another application, Laczka et al. (2014) used an ELISA-type amperometric assay with screen-printed electrodes to detect *Vibrio* species in seawater. The study employed colorimetric detection, with a detection limit ranging from 7 × s10^3^ to 10^4^ cells/mL for most strains, except for *V*. *parahaemolyticus*, which had a lower detection limit of 7 × 10^3^ cells/mL. This species was then targeted for amperometric detection, where the detection limit was improved to 4 × 10^2^ cells/mL, ten times better than colorimetric detection. These studies highlight the widespread application of amperometric immunosensors for pathogen monitoring in various water sources, offering a reliable detection method due to their sensitivity, selectivity, and rapid response time—critical features for accurate pathogen monitoring in water. Wu et al. (2010) developed an amperometric immunosensor modified with zirconium dioxide nanoparticles (ZrO_2_) for detecting *E*. *coli* in polluted water. The three-electrode system included a glassy carbon electrode (GCE) modified with carbon nanotubes (CNTs), ZrO_2_ nanoparticles, colloidal gold (Au), and poly-Lysine (pLL) complexes. Anti-*E*.* coli* antibodies were immobilized on the GCE complex, ensuring the stability of the immunoreaction over time. The immunosensor maintained similar activity to its initial performance after 30 days of storage at 4 °C, with intermittent testing every 3 to 5 days. It was used to detect *E*. *coli* in river water, with a detection limit of 45.5 ng/mL, lower than the traditional ELISA method, which detected *E*. *coli* at 49.8 ng/mL. This immunosensor demonstrated sensitivity, accuracy, and stability, offering advantages over traditional molecular methods. Vásquez et al. (2017) developed a single biotinylated antibody amperometric sensor for detecting *Streptococcus agalactiae* in fish and environmental water samples. The biosensor utilized a single biotinylated antibody cast on a screen-printed carbon electrode (SPCE), conjugated with streptavidin–horseradish peroxidase to generate an amperometric signal. This immunosensor was able to detect *S. agalactiae* in a concentration range of 10^1^ to 10^7^ CFU/mL, with the detection limit reaching 10^1^ CFU/mL. The biosensor showed specificity for *S*. *agalactiae*, with a low signal generated when co-existing species were tested. This development demonstrated that the biosensor has potential applications not only in environmental monitoring but also in food quality and clinical analysis, where *S*. *agalactiae* can be a dominant species.

#### Voltammetric immunosensors

Voltammetric-based sensors, such as cyclic voltammetry, square wave voltammetry, and differential pulse voltammetry, have been effectively employed for pathogen detection in water [3]. These sensors can be integrated with various biorecognition elements to achieve specific analyte detection. The choice of biorecognition element depends on several factors. Voltammetric biosensors, especially when combined with immunosensors, are widely used in various applications. These sensors utilize voltammetric transducers with antibodies as recognition molecules, targeting specific antigens in a sample (Felix et al., 2018). In a study by Jiang et al. (2013), a solid-state voltammetry-based electrochemical immunosensor was developed to detect *E*. *coli*. The immunosensor featured a three-electrode system modified with gold (Au), platinum wire, and silver/silver-chloride (Ag/AgCl). The antibodies for *E*. *coli* (anti-*E*. *coli* polyclonal antibody) were immobilized on graphite oxide-Ag (P-GO-AG) nanocomposites. The incorporation of AuNPs not only facilitated an efficient platform for antibody (Ab) immobilization but also enhanced the electron transfer rate. The antibodies displayed high specificity toward *E*. *coli*, showing minimal interaction with non-target bacteria. This voltammetric immunosensor demonstrated sensitivity, with a limit of detection of 10 CFU/mL for *E*. *coli*. It was further tested for environmental monitoring and successfully detected *E*. *coli* in lake water with a concentration range of 248–340 CFU/mL. The sensor’s sensitivity and specificity make it a valuable tool for detecting pathogenic microorganisms in water.

In another study by Thiruppathiraja et al. (2011), a voltammetric-based sandwich enzyme-linked immunosensor was developed for detecting *Cryptosporidium parvum*, a pathogenic protozoan. The immunosensor used indium tin oxide (ITO) as the working electrode, modified with dual-labeled gold nanoprobe for detecting *C*. *parvum*. The antibodies for *C*. *parvum* oocysts were immobilized on the gold nanoprobe for biorecognition. This immunosensor was highly specific to *C*. *parvum*, showing no cross-reactivity with other waterborne pathogens. It was also highly sensitive, with a limit of detection of 3 oocysts/mL and a detection range of 5–100 oocysts/mL. Oloketuyi et al. (2020) developed a cyclic voltammetry (CV) immunosensor using a three-electrode system for detecting *Alexandrium minutum*, a type of algae. The working electrode, a glassy carbon electrode, was coated with gold nanoparticles (AuNPs). The voltammetric immunosensor demonstrated high sensitivity for detecting *A*. *minutum* within a linear range of 10^3^ to 10^9^ cells/L, with a detection limit of 3 × 10^3^ cells/L. It also exhibited specificity toward *A*. *minutum*, with no response when other microalgae were tested. This sensor could detect *A*. *minutum* in seawater and brackish water without the need for sample concentration, offering several advantages over traditional detection methods. It is cost-effective and suitable for real-time monitoring without interference from other biological contaminants. Wang et al. (2019) developed a Faraday cage-type immunosensor that combined electrochemiluminescence (ECL) and anodic stripping voltammetry (ASV) for detecting *Vibrio parahaemolyticus*. The dual detection methods validated each other’s ability to detect the pathogen, both achieving a detection limit of 33 CFU/mL. The sensor demonstrated a linear response, with signal intensity increasing as the concentration of *V*. *parahaemolyticus* increased, ranging from 10^2^ to 10^7^ CFU/mL. This immunosensor showed high specificity for *V*. *parahaemolyticus*, as no signal response was observed when other bacterial strains were tested. When applied to real water samples, the dual-detection mode demonstrated accuracy, with similar results obtained from both detection methods.

#### Impedimetric immunosensor

Impedimetric biosensors differ from amperometric and potentiometric systems in that they operate through a non-label detection process, meaning they do not require a specific enzyme for biorecognition to occur (Amini & Kraatz, 2015; Leva-Bueno et al., 2020). In a study by Kim et al. (2015), a dielectrophoretic impedance system was used to detect *E*. *coli* in drinking water, with the lowest concentration quantified at 300 CFU/mL within a minute. The device demonstrated high sensitivity for microorganism detection in drinking water. Impedimetric biosensors have proven effective for assessing water quality by monitoring microbial activity, providing rapid, low-cost pathogen detection. In a study by Cimafonte et al. (2020), an impedimetric immunosensor was developed for the rapid detection of *E. coli* in drinking water. The immunosensor was fabricated by immobilizing anti-*E*. *coli* antibodies on the surface of screen-printed gold electrodes using a photochemical immobilization technique, ensuring the antibodies were positioned upright and their binding sites remained exposed. This method proved effective even on low-cost commercial electrodes, making the sensor affordable for widespread use. The sensor detected *E*. *coli* at concentrations as low as 3 × 10^1^ CFU/mL in spiked drinking water, with high specificity toward *E*. *coli* compared to *Salmonella enteritidis* and *Acinetobacter baumannii*. The sensor had a rapid detection time of one hour, offering a significant advantage over traditional methods, which can take days. Mutreja et al. (2016) developed a novel impedimetric immunosensor for detecting the OmpD protein on *Salmonella typhimurium* cells in water. The sensor was based on a screen-printed electrode surface modified with a reduced graphene-graphene oxide (rG-GO) nanocomposite, providing a sensitive recognition surface. The sensor detected *S*. *typhimurium* at concentrations as low as 10^1^ CFU/mL and showed a consistent increase in the impedimetric signal as the cell concentration increased. It also demonstrated high specificity for *S. typhimurium*, with no cross-reaction to non-targeted bacterial species. This immunosensor was successfully tested on water and juice samples, indicating its potential for pathogen monitoring in food analysis.

Escamilla-Gómez et al. (2009) assessed two label-free electrochemical impedance immunosensors for detecting and quantifying *E*. *coli*. Both sensors involved immobilizing antibodies on self-assembled monolayers (SAM) on gold screen-printed electrodes (AuSPEs) using different cross-linkers. The performance of both sensors was monitored by measuring changes in resistance in the presence of the [Fe (CN)6]3-/[Fe (CN)6]4- redox probe. While both sensors were capable of detecting *E*. *coli*, the thiolated immunosensor exhibited better analytic performance, with a detection range of 5 to 1.0 × 10^8^ CFU/mL and a detection limit of 3.3 CFU/mL. The sensor showed excellent specificity toward *E*. *coli*, with minimal cross-reaction when tested against *Staphylococcus aureus* and *Salmonella* *choleraesuis*. The thiolated immunosensor was further tested on river and tap water samples, detecting *E*. *coli* at concentrations of 12 ± 2 CFU/mL in river water and 11 ± 2 CFU/mL in tap water. This immunosensor demonstrated higher sensitivity and quicker detection (1 h) compared to traditional microbiological methods, with no need for sample pretreatment. In a study by Bekir et al. (2015), an impedimetric immunosensor was developed by immobilizing polyclonal antibodies on a screen-printed carbon electrode modified with poly (pyrrole-3-carboxylic acid) (P3CA) to detect *Pseudomonas aeruginosa* in water. The sensor showed a linear increase in the impedimetric signal as the concentration of *P*. *aeruginosa* increased, within a range of 10^1^ to 10^7^ CFU/mL. The sensor was highly specific to *P*. *aeruginosa*, with no significant signal changes observed for non-target bacterial species. This rapid detection method can be effectively applied to real water samples, providing early warnings about waterborne pathogens and helping to prevent disease outbreaks. Impedimetric immunosensors are useful tools for detecting low concentrations of various pathogens, thus enabling early detection of potential health threats. These studies demonstrate the wide applicability and advantages of impedimetric biosensors for pathogen detection in water, including rapid analysis, high sensitivity, specificity, and low-cost implementation. Table 2 summarizes various electrochemical detection systems for waterborne pathogens.

**Table 2:** Electrochemical biosensing system for detection of waterborne pathogens

Signal transduction method	Biorecognition element	Organisms	Linear range (CFU/mL)	LOD (CFU/mL)	Response time (h)	Ref
Amperometric	Antibodies	*Vibrio cholera* O1		20	0.92	Rao et al. (2006); Ramanujam et al. (2021)
	None	*E*. *coli*	10^4^–10^9^	6.40 × 10^4^	0.000139	Ramanujam et al. (2021)
Voltammetric	Antibody magnetic beads Polyguanine (PolyG) polystyrene beads	*E*. *coli* O157:H7	3–3 × 10^2^	3	2	Jayamohan et al. (2015)
Impedimetric	None	*E*. *coli* O157:H7	10^3^–10^8^	10^3^	1	Wu et al. (2020)
Voltammetric	Antibodies	*E*. *coli*	10 × 10^4^	20		Beeman et al. (2018)
Voltammetric	β-galactosidase	*E*. *coli*	10^3^–10^7^	10^3^	4	Bigham et al. (2019)

### Optical biosensors

The biosensing process using an optical transducer detects biomolecule-analyte recognition events by monitoring changes in the medium’s light properties, such as absorption, transmission, reflection, refraction, or fluorescence (Pashazadeh et al., 2017). A study conducted by Wu et al (2021) have shown a concept of incorporating the whole cell, *E*. *coli* into a biosensor system, creating a whole cell biosensor generating a green fluorescence signal. This biosensor functioned by detecting the two waterborne pathogen, *Pseudomonas aeruginosa* and *Burkholderia pseudomallei* through a quorum sensing mode. The *E*. *coli* cells were engineered with plasmids carrying quorum sensing module coupled with reporting module that gave green fluorescence signal when the targeted pathogens were detected and reporting module.

The adoption of optical biosensors has recently grown due to their advantageous features. These biosensors are highly favored for their ease of use, portability, high sensitivity, and rapid analyte detection capabilities (Ali et al., 2020; Dey & Goswami, 2011). They represent a promising technology for the quick detection of pathogens (Amini & Kraatz, 2015). Optical biosensors can utilize various transduction methods, including surface plasmon resonance, Raman spectroscopy, FTIR, colorimetric techniques, and fiber optics (Willner & Vikesland, 2018).

#### Surface plasmon resonance immunosensor

Surface plasmon resonance (SPR) detects target analytes by measuring changes in the refractive index on a metal surface, typically modified with probe molecules (Pires et al., 2014). Recent advancements have highlighted SPR's potential as a widely adopted sensing method due to its ability to enable rapid, label-free detection of various antigens (Torun et al., 2012).

**Fig. 4:**
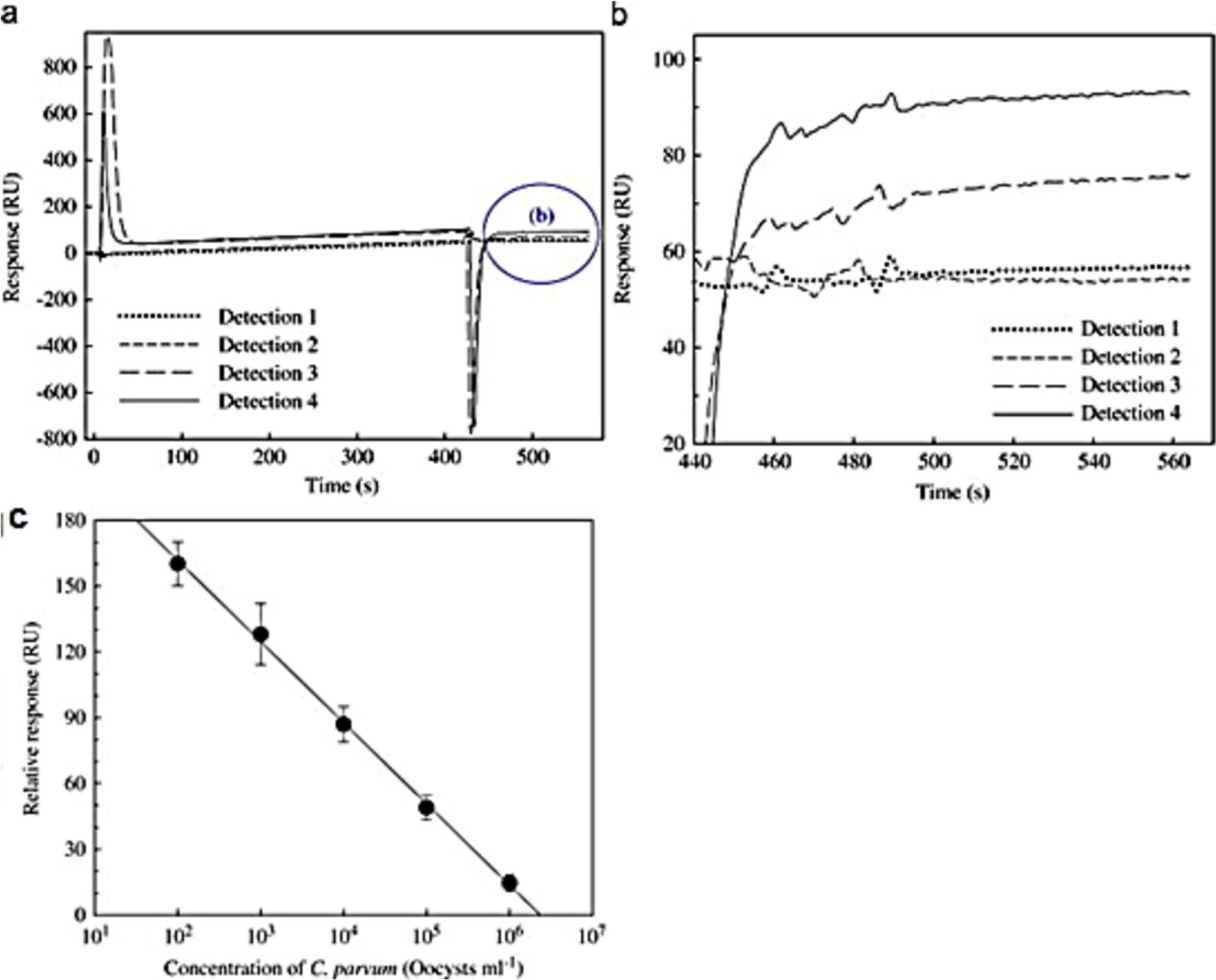
**a**, **b** Performance tests of the *Cryptosporidium* sensor chip during SPR-based inhibition assay. Detection 1: *C. parvum* oocyst in HBS-EP buffer; Detection 2: mixture of *Bacillus stearothermophilus* spore, *Chlamydomonas reinhardtii*, *Escherichia coli*, and *C*. *parvum* oocyst in HBS-EP buffer; Detection 3: *C*. *parvum* oocyst in tap water; and Detection 4: *C*. *parvum* oocyst in reservoir water. The concentrations of the used microorganisms were 1 × 10^5^ cells ml^−1^ in all cases. **c **Detection range of *C*. *parvum* oocysts in the SPR-based inhibition assay method using the anti-mouse IgM-arrayed *Cryptosporidium* sensor chip (*R*.^2^ = 0.9987, *n* = 5) (Kang et al., 2008)

In one study, Foudeh et al. (2015) developed an SPR imaging (SPRi) technique using rRNA as a receptor to monitor *Legionella pneumophila* in water samples, particularly in the presence of protozoa. This method detected *L*. *pneumophila* in concentrations ranging from 3 × 10^4^ to 3 × 10^8^ CFU/mL^−1^ within 3 h, showing high specificity as the SPR signal was observed only for *L*. *pneumophila* compared to other bacterial species. Similarly, Manera et al. (2013) developed an SPR immunosensor using a polyclonal antibody for *L*. *pneumophila* detection in water samples. The immunosensor demonstrated high selectivity and detected the pathogen within one hour. Dudak and Boyaci (2007) designed an SPR immunosensor to detect *Escherichia coli* in water samples at concentrations ranging from 9.0 × 10^1^ to 1.8 × 10^5^ CFU/mL. The sensor’s surface was regenerated with 1% SDS without loss of binding efficiency. It also exhibited specificity when tested against *Enterobacter aerogenes* and *Enterobacter dissolvens*, showing a stronger response to *E*. *coli*. Applied to real water samples, the sensor delivered results comparable to plate counting methods, offering advantages such as sensitivity, selectivity, and reusability. SPR immunosensors have also been employed to detect *Vibrio cholerae*. Kang et al. (2008) and Taheri et al. (2017) developed a specific SPR immunosensor targeting the OmpW protein of *V. cholerae*. Anti-OmpW antibodies immobilized on a chip detected antigens in a concentration range of 10^1^ to 10^8^ cell mL^−1^, with a detection limit of 50 cells/mL. The sensor demonstrated high selectivity against other bacterial species and could regenerate binding efficiency, providing rapid, sensitive, and specific detection without requiring sample pre-treatment. In another study, Kang et al. (2008) developed an SPR inhibition assay to detect *Cryptosporidium parvum* oocysts. The sensor utilized modified chips with biotin, streptavidin, and biotinylated secondary antibodies. Detection was achieved by measuring free unbound monoclonal anti-*C*. *parvum* antibodies, with signals inversely proportional to oocyst concentrations. The sensor showed high specificity for *C*. *parvum* but required optimization to improve sensitivity under environmental conditions. Overall, SPR immunosensors have proven effective for microbial risk assessment in water, offering rapid, on-site detection with high sensitivity and specificity. These advancements make them a promising alternative to conventional methods for waterborne pathogen monitoring. Surface plasmon resonance (SPR) technology identifies target analytes by detecting changes in the refractive index on a metal surface modified with probe molecules (Pires et al., 2014). Recent advancements highlight SPR’s growing role as a sensing method due to its ability to facilitate rapid, label-free detection of various antigens (Torun et al., 2012). For instance, Foudeh et al. utilized surface plasmon resonance imaging (SPRi) integrated with rRNA receptors to monitor *Legionella pneumophila* in water samples, particularly those containing protozoa. Their SPRi method detected *L*. *pneumophila* at concentrations ranging from 3 × 104 to 3 × 108 CFU/mL within 3 h. The technique demonstrated specificity, as the SPR signal was observed only for *L*. *pneumophila* and not for other bacterial species. In another study, Manera et al. (2013) developed an SPR-based immunosensor using a polyclonal antibody to detect *L*. *pneumophila* in water. This sensor exhibited high selectivity, distinguishing *L*. *pneumophila* from *E*. *coli* and *Salmonella* spp., and detected the pathogen within one hour. Similarly, Dudak and Boyaci (2007) developed an SPR immunosensor for *E*. *coli* detection. Their sensor operated across a concentration range of 9.0 × 101 to 1.8 × 105 CFU/mL and could regenerate its surface using 1% SDS without losing binding efficiency. When tested against *Enterobacter aerogenes* and *Enterobacter dissolvens*, the sensor exhibited lower response signals for non-target bacteria, affirming its specificity. Moreover, its performance in real water samples was comparable to traditional plate-counting methods but with greater sensitivity, selectivity, and reusability. Kang et al. (2008) and Taheri et al. (2017) applied SPR immunosensors to detect *Vibrio cholerae* via its outer membrane protein, OmpW. Anti-OmpW antibodies immobilized on a modified chip interacted with OmpW antigens at concentrations ranging from 10^1^ to 10^8^ cell mL^−1^ with a detection limit of 50 cells mL^−1^. The sensor’s regeneration buffer restored its binding efficiency for reuse. It demonstrated specificity, producing low detection signals for non-target bacteria, and provided rapid, sensitive, and specific detection without the need for sample pre-treatment. Additionally, Kang et al. (2008) developed an SPR-based inhibition assay for detecting *Cryptosporidium parvum* oocysts. A sensor chip modified with biotin, streptavidin, and antibodies was used to detect unbound anti-*C*. *parvum* antibodies, inversely correlating with oocyst concentration 1 × 10^6^–1 × 10^2^ cell mL^−1^ as shown in Fig. 4c. While the sensor exhibited exclusive sensitivity to *C*. *parvum*, environmental factors reduced its detection capability, necessitating optimization for environmental applications. The sensors performance in different environments was evaluated as shown in Fig. 4 a and b. Sensorgram changes were observed due to variations in the refractive indices of different environments. Cryptosporidium oocysts were reliably detected at a concentration of 1 × 10^5^ oocysts/mL, consistent with prior calibration (Detection 1; control). Detection 2, employing anti-C. parvum oocyst (mouse IgM), demonstrated high specificity, even in the presence of other microorganisms. The assay was effective in natural environments, detecting oocysts in tap (Detection 3) and reservoir water (Detection 4), though with reduced sensitivity. This was likely due to suboptimal conditions for antibody interactions, influenced by factors like pH, ionic strength, and trace elements. Optimizing these environmental parameters could enhance sensitivity. Overall, SPR immunosensors are highly effective for rapid, on-site microbial risk assessment in water, offering sensitivity, specificity, and versatility compared to conventional methods.

#### Chemiluminescence

Chemiluminescence detects target analytes by generating photochemical emission during biorecognition events. In a study by Pires and Dong (2015), a chemiluminescence immunosensing system was developed for the detection of multiple pathogens in water. This system utilized a microfluidic chip composed of poly (methyl methacrylate) (PMMA) material, featuring an array of capillary-induced flow microchannels and chambers. The chip was equipped with eight ring-shaped organic photobodies and further enhanced with gold nanoparticles integrated into the microfluidic chambers. The device detected *E*. *coli* at a detection limit estimated around 6.4 × 10^4^ cells mL^−1^. The system also demonstrated its capability for multiplex detection of *E*. *coli*, *Campylobacter jejuni*, rotavirus, and adenovirus by functionalizing the chambers with antibodies specific to each targeted pathogen. Each chemiluminescence test chamber was uniquely designed to detect its corresponding pathogen without cross-reactivity. The study highlighted the device’s potential for monitoring waterborne pathogens, offering simultaneous detection of multiple targets without interference. This multiplex capability provides comprehensive pathogen analysis in a single assay, reducing effort and time compared to traditional single-pathogen detection methods. Its high sensitivity further underscores its value as an advanced technology for water analysis. Similarly, Wolter et al. (2008) developed a chemiluminescence biosensing system for detecting *E*. *coli*, *Salmonella typhimurium*, and *Legionella pneumophila*. This system utilized a chemiluminescence microarray created by surface modification of glass slides, which were activated and functionalized with polyethylene glycol to minimize cross-reactions and facilitate antibody-antigen interactions. The slides were further modified to achieve an N-Hydroxysuccinimide (NHS)-activated surface, allowing covalent immobilization of species-specific antibodies. This chemiluminescence sandwich ELISA sensor exhibited precise and specific detection of target pathogens, completing the assay within just 13 min in buffer, demonstrating its rapid detection capability. The sensor was also able to detect *S*. *typhimurium*, *E*. *coli*, and *L*. *pneumophila* in a single reaction with a detection limits of 3 × 10^6^ cells mL^−1^, 1 × 10^4^ cells mL^−1^, and 1 × 10^5^ cells mL^−1^ respectively. The antibodies used in this sensor were highly specific to their target species, with no cross-reactivity observed during simultaneous detection. This chemiluminescence microarray immunosensor demonstrated several advantages over conventional microbiological methods, including sensitive and selective detection within a short timeframe. Additionally, its ability to detect multiple targets in a single sample makes it a convenient and efficient technique for water analysis. The application of sensitive chemiluminescence immunosensors for detection of *E*. *coli* O157:H7 in various consumable liquid samples including water was demonstrated in a study done by Zhang et al. (2014). The developed chemiluminescence immunosensors demonstrated its ability to detect *E*. *coli* O157:H7 at a concentration level of 1.2 × 10^3^ CFU/mL, which was remarkably lower than that of ELISA at 1.0 × 10^5^ CFU/mL. Chemiluminescence signal intensified with increasing concentration of *E*. *coli* O157:H7 without pre-treatment of samples and detection was done in less than 2 h. This sensor also demonstrated high specificity when closely related species to the targeted species were also tested, there was no significant chemiluminescence signal compared to signal observed when of *E*. *coli* O157:H7 was tested. These positive analytic results were further supported by application of this sensor in analysing real water samples and consumable liquid samples which showed similar quantities of those obtained when ELISA was applied. Therefore, this technique can be used rather than ELISA because it has shown sensitivity, specificity and shorter detection time which are the crucial factors in assessing water quality in preventing disease manifestation.

#### Colorimetric immunosensors

Detection of target pathogen can be easily done by the application of colorimetric biosensors. Park et al. (2010) used colorimetric and chemiluminescence optical methods coupled with an immunostrip system to detect *Salmonella typhimurium* in river water. These two optical methods successfully detected *S*.* typhimurium* at a concentration range of 10^3^ and 10^9^ CFU/mL within 20 min. The system of colorimetric gives a colour change when an analyte is detected which can be observed by the naked eye (Bhatt & Bhattacharya, 2019). Kim and Yeo (2016) developed a microfluidic paper-based analytical device (μPAD) for rapid colorimetric detection of *E. coli* from the environmental water sample and the results showed that the colour density of μPAD increased with decreasing *E*. *coli* concentration ranging from 0 cells mL^−1^ to 8.77 × 10^8^ cells mL^−1^. In another study by Ranjbar et al. (2019), an electrochromic immunosensor developed on the polyalanine (PANI) and indium tin oxide (ITO) screen-printed electrodes was applied in the detection of *E*. *coli* in water samples. The mechanism of the immunosensor was based on colour change (from yellow to blue) when the constant potential was applied, therefore being a blend of an optical and electrochemical sensor. Different antibody concentrations (2.5, 5.0, and 10 µg mL^−1^) were used for optimizing the functionality of the immunosensor, which play a crucial factor in capturing the analyte. The smallest and highest concentration did not show efficient binding capabilities, while the middle concentration showed optimum binding capabilities. Another important factor in the developed electrochromic sensor is the choice of the applied potential because it determines the detection range and sensitivity of the sensor. The sensor took 60 min to give out an output of the sensing system. The results of the study showed the electrochromic immunosensor was able to detect bacteria at 10^2^ CFU/mL with a naked eye, while a concentration of 10^1^ CFU/mL was detected when using ImageJ software. The sensor also showed different colour changes when *Salmonella typhimurium* was tested as compared to the colour observed when *E*. *coli* was tested, therefore the used antibodies were specific to *E. coli* only. The sensor was further applied for the detection of *E. coli* in water and had shown the ability to detect contaminants in water. This electrochromic sensor showed several advantages such as its ability to perform real-time detection, on-site application, sensitivity, and ease of use. Laczka et al. (2013) detected *Cryptosporidium parvum* oocysts by application of an ELISA-type screen printed electrode-based potentiometric detection. The *Cryptosporidium* oocysts capture and detection was first optimized by colorimetric detection based on the traditional ELISA plate system. The colorimetric limit of detection was observed at 1 × 10^3^ oocysts per mL; however, the antibody binding sites were saturated at a concentration of 1 × 10^6^ oocysts per mL. From this study, it was observed that the performance of colorimetric immunoassay was 100 to 1000 times that of traditional ELISA. Once the detection was optimised the assay was subjected to potentiometric detection involving screen-printed electrodes. The detection times took 60 min when the electrodes were functionalized with the capture antibodies. The two techniques were compared for the sensitivity, where the limit of detection of oocysts was 1 × 10^3^ oocysts per mL and 5 × 10^2^ oocysts per mL for colorimetric and potentiometric respectively. Amin et al. (2020) developed an innovative colorimetric immunosensor system that incorporated Förster resonance energy transfer (FRET) coupled with gold nanoclusters (AuNCs) as a signal detector for the detection of *E*. *coli* O157:H7 in a microtube. The FRET detecting system involved the gold nanoparticles (AuNPs) as energy acceptors and AuNCs as energy donors, therefore generating fluorescence light (off/on effect) while in the vicinity of each other. This emission allowed observation of assay with the naked eye and the quantification was obtained using a smartphone camera which produced pictures that were analysed with the ImageJ app. The app converted the brightness into a numerical value. The sensor was able to detect *E*. *coli* at a limit of detection of 4.0 CFU/mL. The AuNPs were an immobilization platform for anti-*E*. *coli* antibodies which were specific to *E*. *coli*; this was observed when the results of 10^5^ CFU/mL *S*. *typhimurium* and 0 CFU/mL *E*. *coli* were similar which showed low brightness due to the “turn off” FRET effect. This developed biosensing system has demonstrated multiple advantages such as sensitivity and specificity. It also allows for the possibility to conduct on-field pathogen detection, less bulky instrument, it is easy to use, uses smartphone which is a familiar device to most world population and the results are easy to interpret therefore does not require a trained person to operate. This sensor also presents a less costly pathogen detection with high accessibility to low setting communities.

#### Fiber optics immunosensor

Fiber optics have been developed and applied for the detection of pathogens in water. Kramer et al. (2007) developed an automated fiber optic-based biosensor to detect *Cryptosporidium* oocysts; this organism was detected at a concentration of 10^5^ oocysts per mL. Fiber optics biosensors have been reported for several advantages which include simplicity in operation, can detect multiple analytes and can be miniaturized (Mehrvar et al., 2000). The principle of this optical method is based on electromagnetic waves which spread through the optical fiber by the reflection of light at the sensor surface (Lim, 2003). In a study conducted by Angelopoulou et al. (2021), a fiber optic technique referred to as white light reflectance spectroscopy (WLRS) immunosensor was developed and applied for detection of *Salmonella typhimurium* in drinking water. Experimentally, the immunosensor detected *S*. *typhimurium* lipopolysaccharides (LPS) and the pathogen. Analytically, the immunosensor has shown to detect LPS and *S*. *typhimurium* in a working range of 10–1000 ng mL^−1^ and 600–5 × 10^7^ CFU/mL with the detection limits reached at 10 ng mL^−1^ and 600 CFU/mL respectively. In comparison with traditional ELISA, the detection limit of LPS was 10 times higher and the same detection limit of *S*. *typhimurium* was observed in both. The total assay was performed within 15 min as compared to the lengthy ELISA assay. The WLRS immunosensor was tested for its efficiency in water; it has shown great recovery value. It has shown to be highly specific, due to non-reactivity when tested against closely related species of *S*. *typhimurium* and *E. coli* which is commonly found in contaminated water. Another advantage of this WLRS immunosensor is that it can be regenerated without loss of activity and have shown long term stability, therefore, making it more economically attractive. This is feasible in the analysis of water quality because is a simple technique to perform rapid detection within a short period and due to its small size, it can be used for on-field detection. Additionally, it can be used for food quality analysis and clinical diagnosis for food-related illnesses. This technique has been successfully applied in performing immunoassays during analyte detection. Monoclonal antibody assay fiber optic-based biosensor was used to detect *Salmonella* in irrigation water (Kramer & Lim, 2004). In the study, it was found that the biosensor was able to detect *Salmonella* at a concentration of more than 10^5^ CFU/mL. Fiber optic biosensing system coupled with immunoassay was used in a study for detection of *Enterococci* spp., the organism was detected at a concentration of > 105 CFU/100 mL (Leskinen & Lim, 2008). The results also showed that this method was time-efficient, by reducing the detection time to 2.5 h as compared to the 24 h of traditional methods. In a study by Yildirim et al. (2013), a portable and reusable optical fiber immunosensor biosensors were developed for the detection of adenovirus in wastewater samples. The results of the study have shown that after 5th experiment the signal decreases to less than 80%, which means the sensor surface is no longer effective to give reliable results. Therefore, the developed sensor can be used for 5 times experiment, to obtain repeatable accurate results. The sensor showed specificity to adenovirus when tested with other pathogenic microorganisms in water. The detection range of the sensor was 100 CFU/mL to 2000 CFU/mL and the sensor was able to give out results in less than an hour as compared to traditional methods. The developed sensor showed several advantages that cannot be attained when using traditional methods for pathogen detection in water. The specificity, sensitivity, portable, and reusable characteristics make this sensor an attractive tool in environmental analysis because on-site detection can be achieved in a short period with a great output of pathogen monitoring in water. The reusable trait is good, to allow multiple experiments which give out accurate results. As illustrated in Table 3, several optical biosensing tools for waterborne pathogens are analyzed.

**Table 3:** Optical biosensors for detection of waterborne pathogens

Transductional Signal	Biorecognition element	Nanoparticles	Organism	Linear range (CFU/mL)	LOD (CFU/mL)	Response Time	Ref
Fluorescence	Enzyme β-D-glucuronidase		*E*. *coli*	10^1^–10^7^	7	–	Bekir et al. (2015) b
Surface plasmon resonance	Antibodies	Fe_3_O_4_–Au NPs	*E*. *coli*	30–3.0 × 10^4^	3	25 min	Torun et al. (2012)
Colorimetric			*E*. *coli*	2.17 × 10^2^	10^5^–10^6^	5 min	Liao et al. (2020)
Fluorescence	Aptamer	AUNPs	*E*. *coli*	5–10^6^	3	20 min	Jin et al. (2017)
Fluorescent	Aptamer	Molybdenum (IV) disulfidenanosheets (MoS_2_-Ns)	*S*. *typhimurium*	10^4^–10^7^	10	–	Singh et al. (2016)

## Discussion and prospect of pathogen detection methods

The reviewed methods have shown the capability of detecting pathogens in water sources. Thus, all these methods are necessary for assessing microbial risks in water. However, standard microbiological techniques used in monitoring waterborne pathogens have shown several disadvantages from limited sensitivity, non-specificity, time-inefficiency, enormous workload, and inability to deliver on-site detection. All these factors affect the efficiency of adequate risk assessment of water, therefore placing humans and other animals at high risk of contracting diseases. Regardless of the setback associated with microbiological methods, these methods are largely used due to availability and low costs. The availability of molecular methods is another approach for assessing waterborne pathogens, but still, these techniques are expensive, often not available in countries with limited resources. Another disadvantage of molecular methods in less-resourced communities is their high-cost equipment and the need for well-trained personnel. These methods usually take days to give information on microbial quality of water; therefore, they are less efficient in case of emergency crisis to control waterborne diseases outbreaks.

The urgent waterborne disease outbreaks and emerging water pathogens need sensitive technology and continuous improvement of microbiological and molecular methods. These methods can be integrated with novel biosensors or components of biosensors to achieve rapid detection in short response time and less expensive. Biosensors have shown great potential in monitoring pathogens, toxins, pesticides, and metals in water. Therefore, these tools are significant in monitoring risks associated with water and are of great value since it is possible to perform point-of-care analysis using them and are easy to use which can be used by any person without undergoing training. For the future, a rapid detection method with ease of operation, time-efficient, high sensitivity, and point of care use can be possible with the application and improvement of biosensors methods. From this review, it is recommended that biosensors hold the potential as a future technology to detect pathogens in water, food, and for medical diagnosis at a low cost. The technology can be integrated with nanomaterials to afford the sensitivity of biosensors towards low concentrations of contaminants which can even be harmful to humans and aquatic animals. The improvement of biosensor performance can also be achieved by the integration of nano-hybrid particles. While, using traditional methods authorities should go through biosensing technology due to the advantages served by it, especially in controlling urgent water disease outbreaks.

## Summary and conclusion

Waterborne pathogens, introduced through fecal contamination, agricultural runoff, industrial activities, and inadequate water infrastructure, pose significant public health risks by causing diseases like diarrhea, cholera, and typhoid. These pathogens contribute to high mortality rates, especially in children. While traditional culture-based and molecular methods have been widely used for pathogen monitoring, their limitations—such as lengthy detection times, insufficient sensitivity, and high costs—hinder rapid and effective water quality assessments.

Biosensors have emerged as promising alternatives, offering rapid, sensitive, portable, and cost-effective pathogen detection. These tools can identify pathogens at low concentrations and have been applied to detect pharmaceutical pollutants, pesticides, and other contaminants in water and soil. Unlike traditional methods, biosensors provide real-time, point-of-care analyses that are user-friendly and do not require specialized training, making them valuable for on-site testing. Although traditional and molecular methods remain the standard for pathogen detection due to their availability and affordability in some regions, they are often time-inefficient and unsuitable for emergency responses during disease outbreaks. Molecular techniques, such as PCR, NAATs, and LAMP, etc., provide improved sensitivity but are expensive and require skilled personnel, limiting their use in resource-constrained settings. The integration of biosensors with nanomaterials and nano-hybrid particles could further enhance their sensitivity and specificity, making them highly effective in detecting trace amounts of harmful contaminants. This advancement positions biosensors as a vital technology for water quality monitoring, risk assessment, and disease prevention, especially in regions with limited resources. In conclusion, biosensors represent a transformative approach to waterborne pathogen detection. With ongoing advancements, these tools could replace traditional methods, providing rapid, efficient, and accessible solutions for safeguarding public health and ensuring water safety globally.

## Data Availability

No datasets were generated or analysed during the current study.
